# Network pharmacology and molecular docking to elucidate the mechanism of pulsatilla decoction in the treatment of colon cancer

**DOI:** 10.3389/fphar.2022.940508

**Published:** 2022-08-08

**Authors:** Huan Liu, Yuting Hu, Baoyu Qi, Chengqiu Yan, Lin Wang, Yiwen Zhang, Liang Chen

**Affiliations:** ^1^ College of Traditional Chinese Medicine, Changchun University of Chinese Medicine, Changchun, China; ^2^ College of Integration Science, Yanbian University, Yanji, China; ^3^ Anorectal Diagnosis and Treatment Center, Affiliated Hospital of Changchun University of Chinese Medicine, Changchun, China

**Keywords:** pulsatilla decoction, colon cancer, quercetin, network pharmacology, molecular docking

## Abstract

**Objective:** Colon cancer is a malignant neoplastic disease that seriously endangers the health of patients. Pulsatilla decoction (PD) has some therapeutic effects on colon cancer. This study is based on the analytical methods of network pharmacology and molecular docking to study the mechanism of PD in the treatment of colon cancer.

**Methods:** Based on the Traditional Chinese Medicine Systems Pharmacology Database, the main targets and active ingredients in PD were filtered, and then, the colon cancer-related targets were screened using Genecards, OMIM, PharmGKB, and Drugbank databases. Then, the screened drug and disease targets were Venn analyzed to obtain the intersection targets. Cytoscape software was used to construct the “Components–Targets–Pathway” map, and the String database was used to analyze the protein interaction network of the intersecting targets and screen the core targets, and then, the core targets were analyzed using Gene Ontology (GO) and Kyoto Encyclopedia of Genes and Genomes (KEGG) analyses. Molecular docking was implemented using AutoDockTools to predict the binding capacity for the core targets and the active components in PD.

**Results:** Sixty-five ingredients containing 188 nonrepetitive targets were screened and 180 potential targets of PD anticolon cancer were identified, including 10 core targets, namely, MAPK1, JUN, AKT1, TP53, TNF, RELA, MAPK14, CXCL8, ESR1, and FOS. The results of GO analysis showed that PD anticolon cancer may be related to cell proliferation, apoptosis, energy metabolism, immune regulation, signal transduction, and other biological processes. The results of KEGG analysis indicated that the PI3K-Akt signaling pathway, MAPK signaling pathway, proteoglycans in cancer, IL-17 signaling pathway, cellular senescence, and TNF signaling pathway were mainly involved in the regulation of tumor cells. We further selected core targets with high degree values as receptor proteins for molecular docking with the main active ingredients of the drug, including MAPK1, JUN, and AKT1. The docking results showed good affinity, especially quercetin.

**Conclusion:** This study preliminarily verified that PD may exert its effect on the treatment of colon cancer through multi-ingredients, multitargets, and multipathways. This will deepen our understanding of the potential mechanisms of PD anticolon cancer and establish a foundation for further basic experimental research.

## 1 Introduction

Colon cancer is a common malignant tumor of the gastrointestinal tract that originates in the mucosal epithelium of the colon. In recent years, the incidence rate of colon cancer has been increasing year by year, and the incidence rate in young people has increased rapidly (Benson et al., 2018). According to the data, the incidence and mortality rate of human colon cancer are 10.2 and 9.2%, respectively, ranking second in the new incidence and mortality rate of cancer (Siegel et al., 2017; Sung et al., 2021). There are many factors in the development of colon cancer, and its clinical symptoms may not show up in the early stage, but in the middle and late stages, there may be abdominal pain, mucopurulent stool, constipation, intestinal obstruction, lymphatic metastasis, etc. (Gupta et al., 2019). The present treatment for colon cancer is mainly based on surgery, chemotherapy, and biological antibody therapy (Yaghoubi et al., 2020). Related adjuvant therapies are also emerging in an endless stream, mainly fluorouracil, capecitabine, and oxaliplatin but with obvious side effects, such as neutropenia, neuropathy, diarrhea, and drug resistance, as well as other related symptoms (Gelibter et al., 2019). Therefore, it is necessary to develop more effective and safer therapeutic strategies for the treatment of colon cancer that reduce toxic side effects and complications and complement the aforementioned therapeutic limitations.

In Traditional Chinese medicine (TCM), colon cancer is called “*Locked Anus Hemorrhoids*.” Before treating colon cancer, TCM physicians will comprehensively understand the patient’s physical condition according to the patient’s pulse, tongue, symptoms, signs, and relevant modern laboratory examination. The treatment scheme varies for patients with different physical statuses, but the overall treatment is based on clearing heat and dampness, cooling the blood, and detoxification. Pulsatilla decoction (PD) comes from the classical Chinese medicine book “*Shang Han Treatise*.” It is a herbal formula composed of four herbs, namely, Phellodendron, Pulsatilla, Coptis Chinensis, and Cortex Fraxini, which have the effect of clearing heat and dampness, cooling the blood, and detoxification. Contemporary pharmacological research has proven that PD has immune function regulation, bactericidal, anti-inflammatory, antitumor, and other effects (Song et al., 2022). It is commonly used for the treatment of amebic dysentery, ulcerative colitis, bacterial dysentery, etc. (Wang L. et al., 2020; Wang X. et al., 2022). In recent years, our research team has been focusing on the regulatory role of mixed and single components of PD in colon tumor cells.

Pulsatilla saponin, an extract of Pulsatilla in PD, demonstrated its antitumor effect by inducing DNA damage and apoptosis and causing G2 receptor arrest in liver, pancreatic, and colon cancer cells (Liu et al., 2014; Xu et al., 2017). Nexrutine, an extract from Phellodendron, has been shown to exhibit antitumor effects in a variety of cancers, such as prostate, gastric, and pancreatic (Gong et al., 2017; Hussain et al., 2018; Zhuang et al., 2020). Berberine, an extract of Coptis Chinensis, inhibited the pernicious behavior of CRC by targeting the MAPK or TGF-β1/Smad signaling pathways to induce the cell cycle, promote apoptosis, and inhibit the growth factor signaling pathway in cancer cells (Zhao et al., 2021). Its chemical component epiberberine can regulate apoptotic in tumor cells *via* the P53 signaling pathway (Yu et al., 2020). Fraxetin, the chemical component of Cortex Fraxini, has been shown to inhibit prostate cancer. Its chemical composition escin can inhibit the effects of ovarian and colorectal cancer (Wang et al., 2018a; Kenny et al., 2021; Ma et al., 2022).

From the perspective of modern pharmacological studies, the extracted components of individual Chinese herbs in PD are candidates for the treatment of colon cancer; however, because of the multicomponent nature of Chinese herbs, the results of the study of PD in the treatment of colon cancer are not sufficient; thus, we need a more comprehensive research theory to systematically explore the relevant biomechanics of PD anticolon cancer. This study quotes the theory of “Network Pharmacology” put forward by pharmacologist Andrew L. Hopkins (Hopkins, 2008) and the theory of “Correlation between TCM and Biomolecular Network” put forward by Professor Li Shao (Li S. et al., 2020b), an expert of traditional Chinese medicine. As an analytical system based on the combination of pharmacology and systems biology theory, network pharmacology is particularly suitable for TCM with complex composition.

**FIGURE 1:**
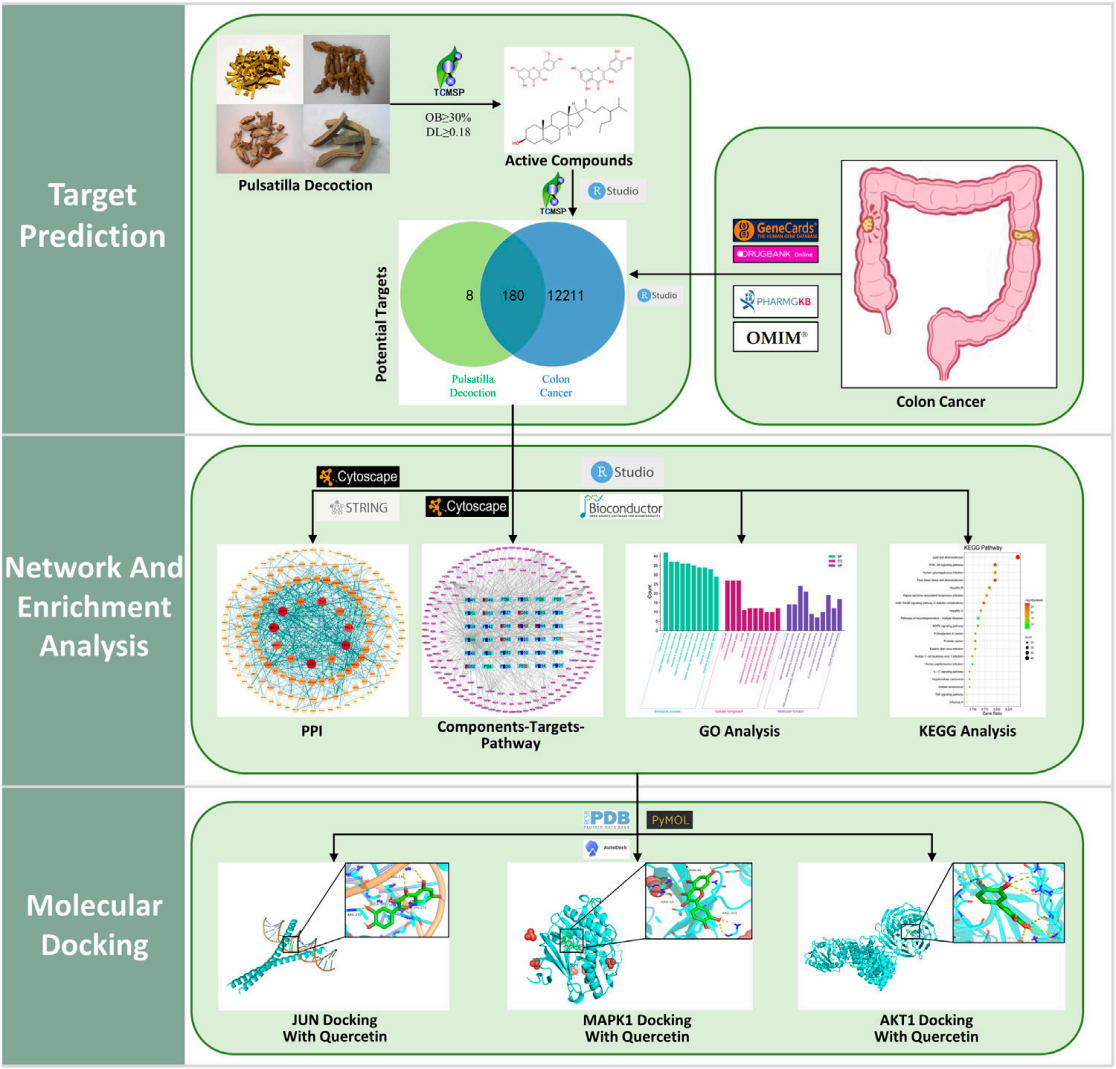
Flow chart of the network pharmacology of pulsatilla decoction (PD) anticolon cancer.

In this research, the method of network pharmacology was applied to construct the “Components–Targets–Pathway” network and validated the core targets and pathways using Gene Ontology (GO) enrichment analysis, Kyoto Encyclopedia of Genes and Genomes (KEGG) pathway analysis, and molecular docking techniques to explore the potential mechanisms of PD anticolon cancer. The flow chart for this study is shown in Figure 1.

## 2 Methods

### 2.1 Screening of components and target prediction of pulsatilla decoction

The keywords “Phellodendron, Pulsatilla, Coptis Chinensis, and Cortex Fraxini” were searched accordingly on the Traditional Chinese Medicine Systems Pharmacology Database (TCMSP) (https://lsp.nwu.edu.cn/tcmsp.php) (Yang et al., 2017; Zeng et al., 2022) to obtain all the chemical components of PD. Moreover, all chemical components were screened for active ingredients according to the screening criteria of bioactive components with oral bioavailability (OB) ≥ 30% and drug-likeness (DL) ≥ 0.18. Using TCMSP to get the potential targets of active components of PD. UniProt database (https://www.uniprot.org/) (Xu X. et al., 2022) was used to limit the species to “Homosapiens” and standardize the annotation of the target genes.

### 2.2 Prediction of colon cancer targets

Colon cancer was searched in the GeneCards (https://www.genecards.org/) (Xiang et al., 2022), OMIM (https://www.omim.org/) (Li et al., 2021), PharmGkb (https://www.pharmgkb.org/) (Zhang et al., 2020), and DrugBank (https://go.drugbank.com/) (Li et al., 2021) databases, respectively, restricted the species to human and selected genes; then, the gene contents of the four databases were combined to remove duplicate values, and the results obtained were the relevant targets for colon cancer.

### 2.3 Target prediction of pulsatilla decoction anticolon cancer

The compound targets of drugs and diseases are the potential targets of drugs acting on diseases (Huang et al., 2022). In this study, the targets of PD and colon cancer were taken to intersect using R language, and the common targets of both are the relevant targets of PD against colon cancer.

### 2.4 Construction of “components–targets–pathway” network

The Cytoscape software (version 3.8.2) was used to correlate the drugs, active components, overlapping targets, pathways, and diseases and construct the interactive network diagram between the components and the targets, in which the edge represents the interrelationship between nodes and the node stands for the active ingredients and targets; then, the main active ingredients of drugs were analyzed.

### 2.5 Screening core targets

The overlapping targets of colon cancer and PD were imported to the String database (https://string-db.org/) (Xu X. et al., 2022; Xiang et al., 2022), and the species was selected as human to obtain protein interaction relationship. The results were exported in TSV format and imported into Cytoscape software to obtain protein–protein interaction (PPI) diagrams, and the CytoNCA plug-in was used to analyze the topology of this network based on the conditions of Betweenness, Closeness, Degree, Eigenvector, Local Average Connectivity, and Network. Then, the importance of nodes in the PPI network was measured based on the classical “degree” value.

### 2.6 Gene Ontology and Kyoto Encyclopedia of Genes and Genomes pathway enrichment analysis

To investigate the biological process and signaling pathway transduction process of PD anticolon cancer, the R language and colorspace, stringi, ggplot2, ClusterProfiler, enrichplot, DOSE, and pathview packages in Bioconductor software were used, and the filtering condition was set to *p* < 0.05 to obtain the enrichment analysis results of GO and KEGG; the results were displayed in barplot or bubble.

### 2.7 Molecular docking

The core target protein screened using PPI network topology analysis was selected as the core receptor protein, and the target protein name was input to the Protein Data Bank (PDB) (http://www.rcsb.org/pdb/) (Tu et al., 2021), and the species was configured with “Homosapiens,” and the high-resolution three-dimensional target protein structure was selected. By using PyMOL software to remove water molecules and original ligand molecules from the PDB file of the original receptor protein, the PDB file of the treated receptor and ligand was imported into AutoDockTools for routine processing and saved as the format file of pdbqt. Then, AutogGrid was used to obtain the docking site parameters, run AutoDockVina for molecular docking, and then take the lowest binding energy as the docking results of the target protein and ligand; PyMOL software was used for analysis and visualization.

## 3 Results

### 3.1 Collection of pulsatilla decoction active components and overlapping targets

**FIGURE 2:**
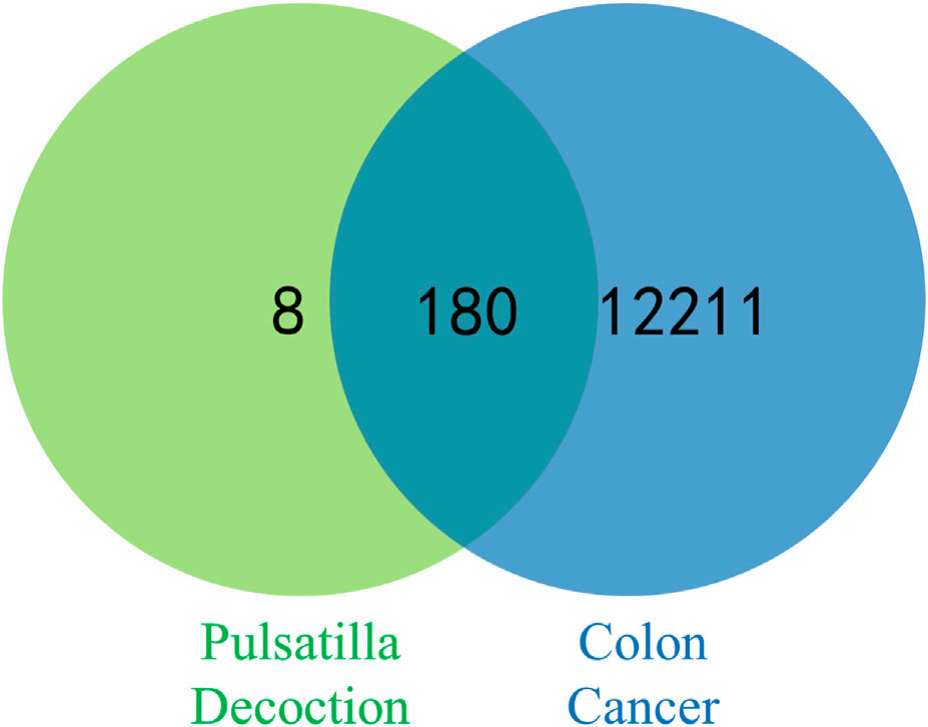
Venn diagram of potential targets for PD against colon cancer.

A total of 65 active ingredients of four drugs in PD were identified by searching the TCMSP under the conditions of OB ≥ 30% and DL ≥ 18%, including 37 Phellodendron, 11 Pulsatilla, 14 Coptis Chinensis, and 3 Cortex Fraxini. Afterward, 53 active ingredients of PD were obtained after removing the repeated ingredients (details in Table 1). Then, 36 active ingredients were obtained after removing the ingredients that were not screened for human-related targets. According to the TCMSP, there were 443 potential targets of active components in PD, including 183 Phellodendron, 66 Pulsatilla, 163 Coptis Chinensis, and 31 Cortex Fraxini. Then, 188 potential targets were obtained after removing the repeated targets. By using GeneCards, OMIM, PharmGkb, and DrugBank databases to integrate data and delete duplicate targets, a total of 12,391 targets linked with colon cancer were identified. Overlapping targets between PD and colon cancer were inputted into the Venn diagram to obtain 180 overlapping targets, which may be potential targets of PD against colon cancer (displayed in Figure 2).

**TABLE 1:** Basic information of active components of PD.

Source	Id	Active ingredient	OB(%)	DL
Cortex Fraxini	PD1	8-betaDGlucopyranosyloxy-7-hydroxy-6-methoxy-2H-1-benzopyran-2-one	36.76	0.42
Cortex Fraxini	PD2	AIDS214634	92.43	0.55
Coptis Chinensis	PD3	Moupinamide	86.71	0.26
Coptis Chinensis	PD4	Corchoroside A_qt	104.95	0.78
Coptis Chinensis	PD5	Berlambine	36.68	0.82
Coptis Chinensis	PD6	(R)-Canadine	55.37	0.77
Coptis Chinensis	PD7	epiberberine	43.09	0.78
Phellodendron, Coptis Chinensis	PD8	Obacunone	43.29	0.77
Phellodendron, Coptis Chinensis	PD9	berberrubine	35.74	0.73
Phellodendron, Coptis Chinensis	PD10	Worenine	45.83	0.87
Phellodendron, Coptis Chinensis	PD11	coptisine	30.67	0.86
Phellodendron, Coptis Chinensis	PD12	berberine	36.86	0.78
Phellodendron, Coptis Chinensis	PD13	palmatine	64.6	0.65
Phellodendron, Coptis Chinensis	PD14	Palmidin A	35.36	0.65
Phellodendron, Coptis Chinensis	PD15	Magnograndiolide	63.71	0.19
Phellodendron, Coptis Chinensis	PD16	quercetin	46.43	0.28
Phellodendron	PD17	thalifendine	44.41	0.73
Phellodendron	PD18	phellochin	35.41	0.82
Phellodendron	PD19	melianone	40.53	0.78
Phellodendron	PD20	dihydroniloticin	36.43	0.82
Phellodendron	PD21	campesterol	37.58	0.71
Phellodendron	PD22	Hispidone	36.18	0.83
Phellodendron	PD23	Hericenone H	39	0.63
Phellodendron	PD24	Candletoxin A	31.81	0.69
Phellodendron	PD25	Cavidine	35.64	0.81
Phellodendron	PD26	Chelerythrine	34.18	0.78
Phellodendron	PD27	Skimmianin	40.14	0.2
Phellodendron	PD28	rutaecarpine	40.3	0.6
Phellodendron	PD29	niloticin	41.41	0.82
Phellodendron	PD30	kihadanin A	31.6	0.7
Phellodendron	PD31	dihydroniloticin	36.43	0.81
Phellodendron	PD32	delta7-Dehydrosophoramine	54.45	0.25
Phellodendron	PD33	Dehydrotanshinone II A	43.76	0.4
Phellodendron	PD34	Phellopterin	40.19	0.28
Phellodendron	PD35	delta 7-stigmastenol	37.42	0.75
Phellodendron	PD36	Phellavin_qt	35.86	0.44
Phellodendron	PD37	Kihadalactone A	34.21	0.82
Phellodendron	PD38	poriferast-5-en-3beta-ol	36.91	0.75
Phellodendron	PD39	(S)-Canadine	53.83	0.77
Phellodendron	PD40	phellamurin_qt	56.6	0.39
Phellodendron	PD41	Isocorypalmine	35.77	0.59
Phellodendron	PD42	Fumarine	59.26	0.83
Pulsatilla, Phellodendron, Cortex Fraxini	PD43	beta-sitosterol	36.91	0.75
Pulsatilla, Phellodendron	PD44	Stigmasterol	43.83	0.76
Pulsatilla	PD45	β-sitosterol	33.94	0.7
Pulsatilla	PD46	ZINC01615307	56.38	0.87
Pulsatilla	PD47	3beta,23-Dihydroxy-lup-20 (29)-ene-28-O-alpha-L-rhamnopyranosyl (1–4) -beta-D-glucopyranosyl (1–6) -beta-D-glucopyranoside_qt	37.59	0.79
Pulsatilla	PD48	LAN	42.12	0.75
Pulsatilla	PD49	Aureusidin	53.42	0.24
Pulsatilla	PD50	Sitosteryl acetate	40.39	0.85
Pulsatilla	PD51	Pulchinenoside C_qt	37.79	0.76
Pulsatilla	PD52	isorhamnetin	49.6	0.31
Pulsatilla	PD53	Mairin	55.38	0.78

OB, oral bioavailability; DL, drug-likeness.

### 3.2 Construction of “components–targets–pathway” network

**FIGURE 3:**
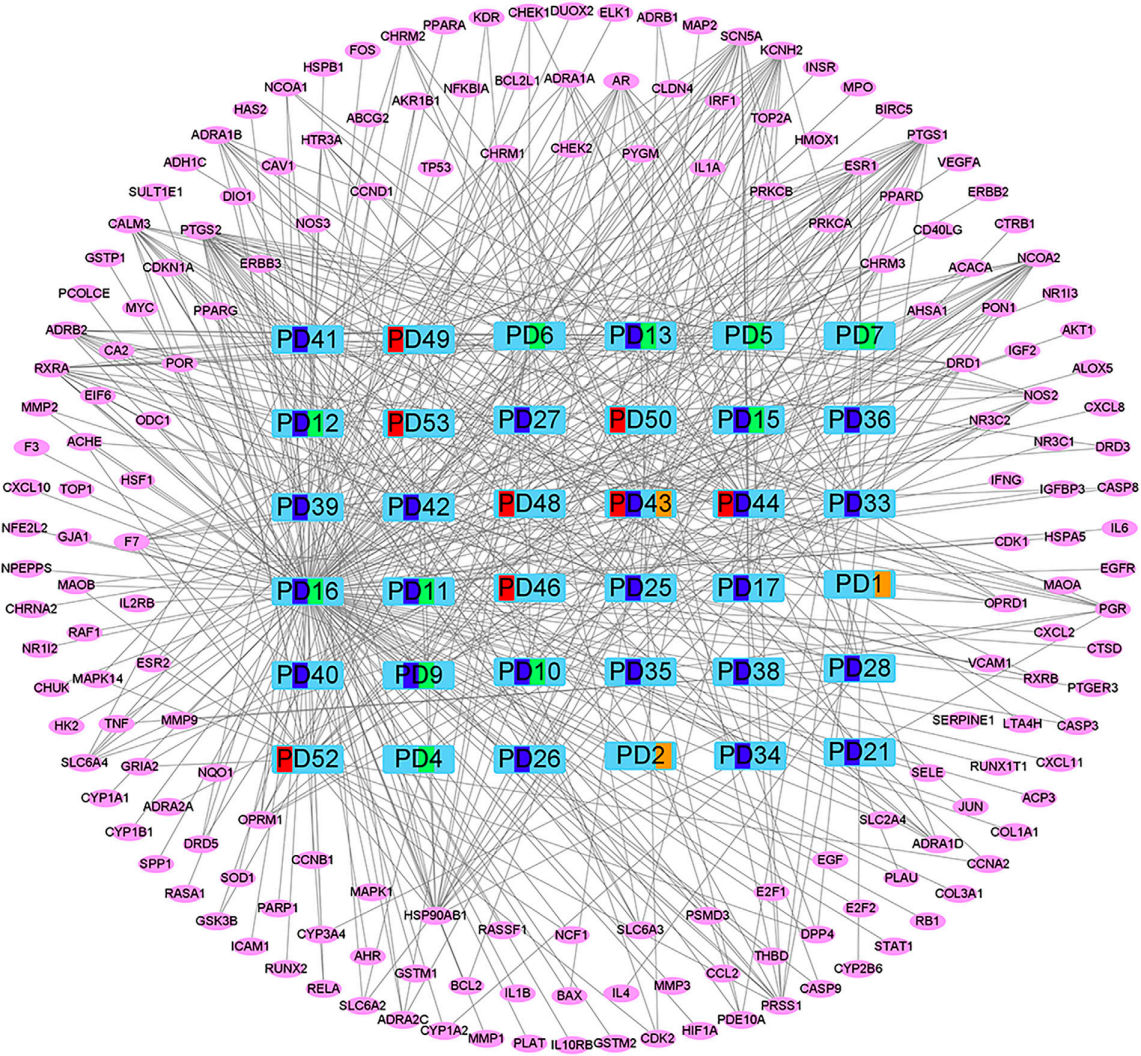
“Components–Targets–Pathway” network. Green represents Coptis Chinensis, dark blue represents Phellodendron, red represents Pulsatilla, orange represents Cortex Fraxini, and pink represents genes.

**FIGURE 4:**
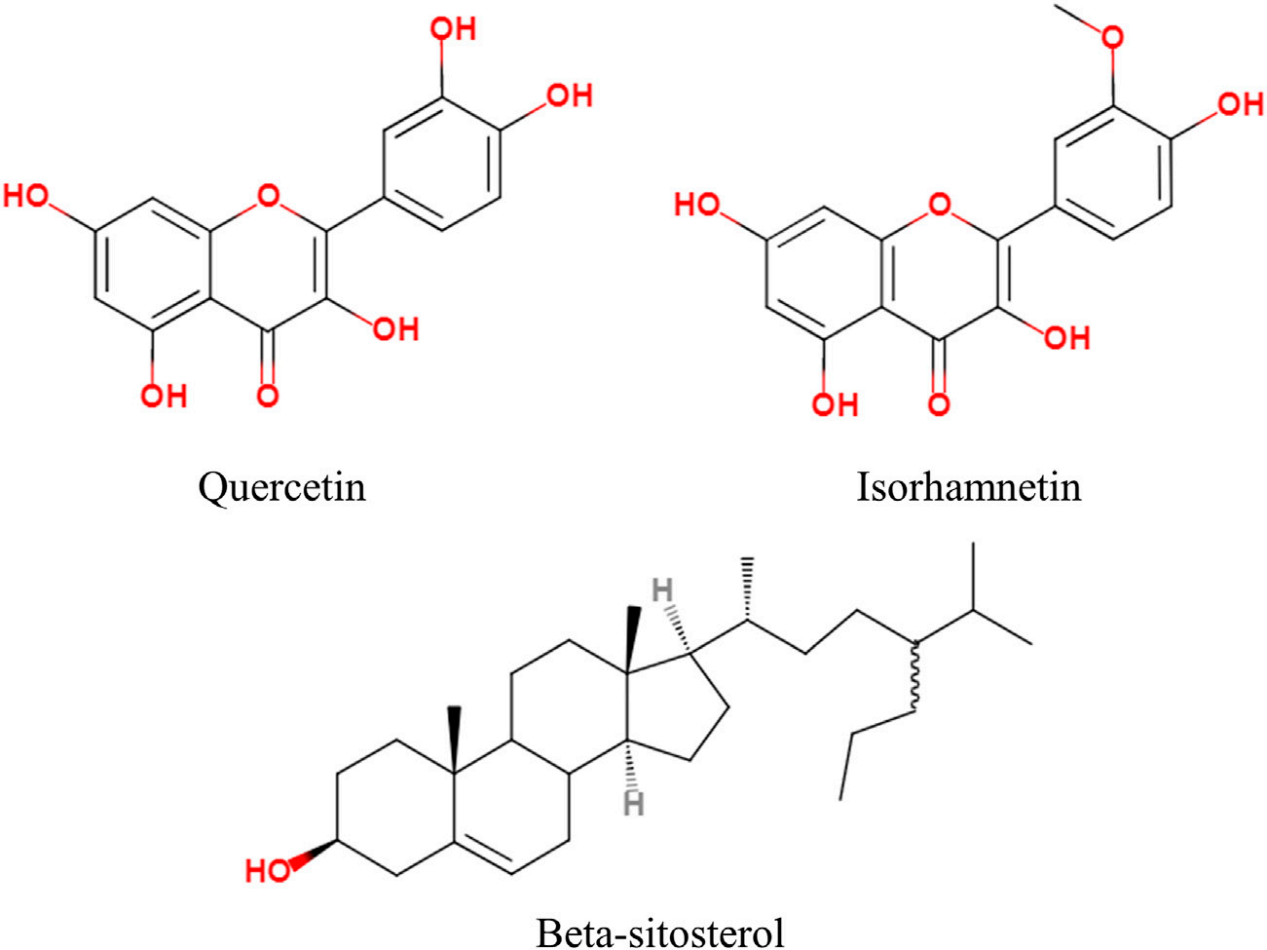
Chemical structure formulas of the top three active components of PD.

The compound targets of Drug and Disease and the active ingredients of drugs were imported into Cytoscape software to construct the “Components–Targets–Pathway” network, which shows the complex relationship between PD and colon cancer. There are 216 nodes and 516 edges in the network, of which green represents Coptis Chinensis, dark blue represents Phellodendron, red represents Pulsatilla, orange represents Cortex Fraxini, and pink represents genes (Figure 3). Among them, quercetin, isorhamnetin, and beta-sitosterol have the highest connectivity, ranking in the top three (structure in Figure 4). Their degree values were 135, 27, and 26, respectively. In the therapy of colon cancer, quercetin may be the most critical component.

### 3.3 Screening core targets

**FIGURE 5:**
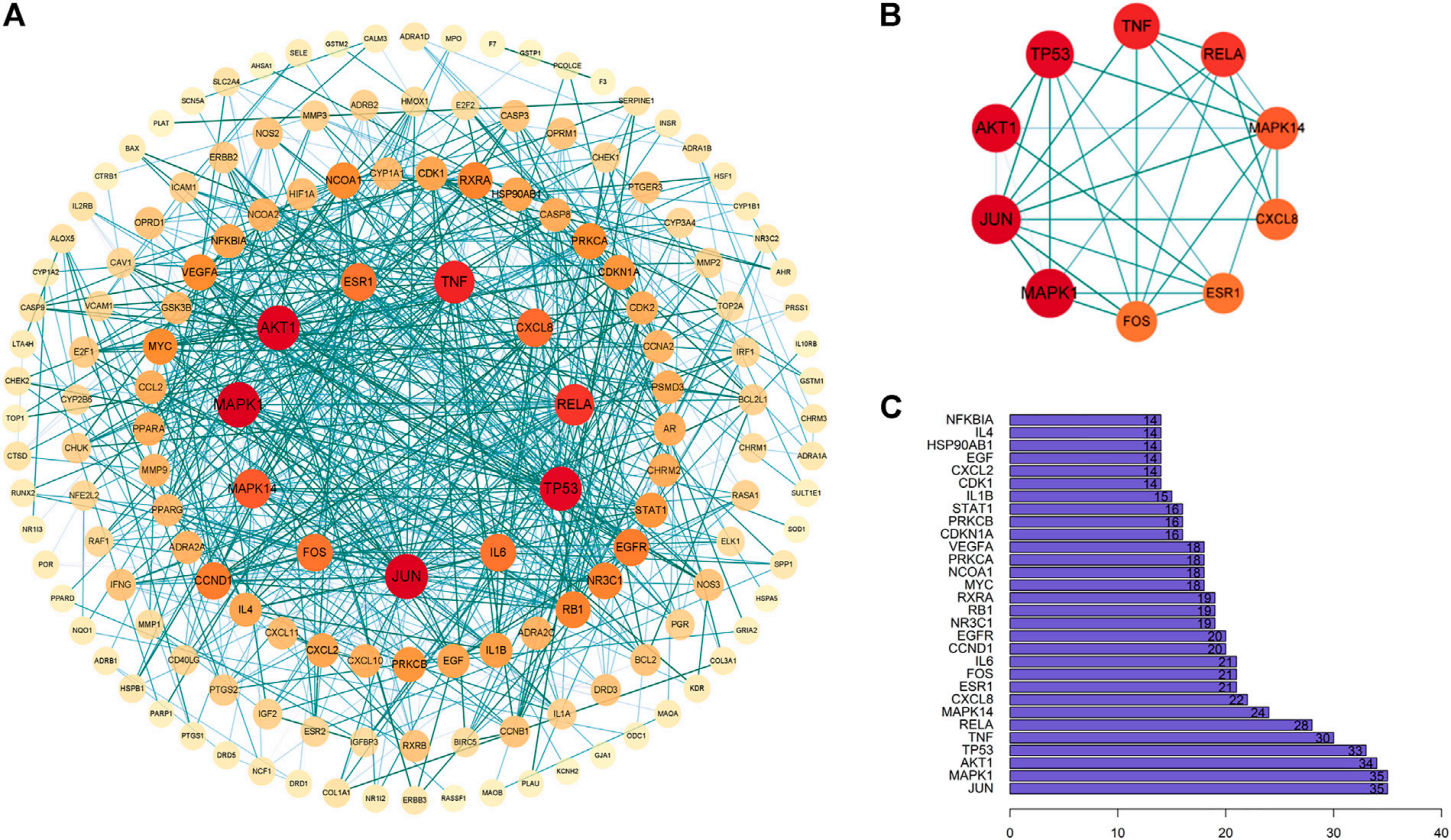
Interaction network diagram of PD target proteins against colon cancer. **(A)** PPI network of 154 duplicate targets. **(B)** network of the top 10 core proteins. **(C)** top 30 targets ranked by the degree value.

The common targets of colon cancer and PD were imported into the String database, and 154 target proteins and 676 protein interactions were obtained. The results were transferred to Cytoscape software to obtain PPI maps and topological analysis (Figure 5). To enhance its reliability, a confidence score of ≥0.9 was set. The results showed that the top 10 core proteins were MAPK1, JUN, AKT1, TP53, TNF, RELA, MAPK14, CXCL8, ESR1, and FOS (Table 2).

**TABLE 2:** Top 10 core proteins information.

Uniprot Id	Sybol	Protein names	Degree
P28482	MAPK1	Mitogen-activated protein kinase 1	35
P05412	JUN	Transcription factor AP-1	35
P31749	AKT1	AKT Serine/Threonine Kinase 1	34
P04637	TP53	Cellular tumor antigen p53	33
P01375	TNF	Tumor necrosis factor	30
Q04206	RELA	Transcription factor p65	28
Q16539	MAPK14	Mitogen-activated protein kinase 14	24
P10145	CXCL8	Interleukin-8	22
P03372	ESR1	Estrogen receptor	21
P01100	FOS	Fos Proto-Oncogene, AP-1 Transcription Factor Subunit	21

### 3.4 Gene Ontology and Kyoto Encyclopedia of Genes and Genomes enrichment analyses

**FIGURE 6:**
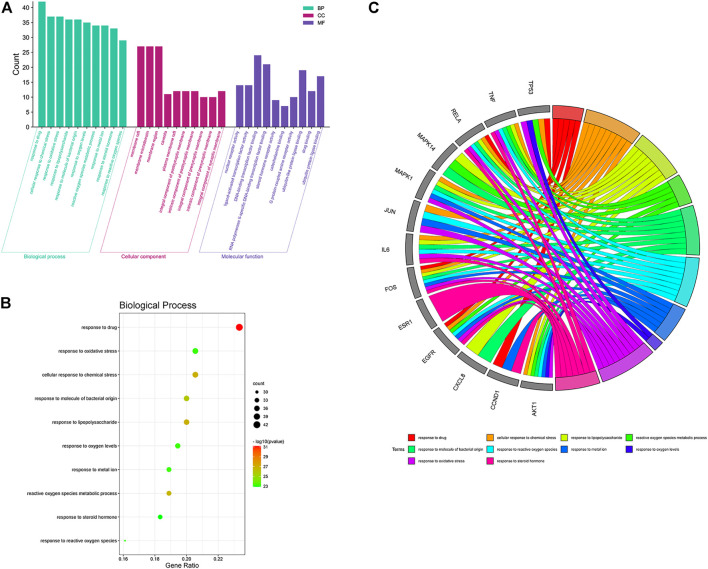
Gene Ontology (GO) function enrichment analysis of PD in the treatment of colon cancer. **(A)** GO function analysis, including BP, CC, and MF. **(B)** bubble diagram of BP enrichment. **(C)** GO chord diagram of the top 10 BP in the PD anticolon cancer.

To further investigate the mechanism of the anticolon cancer effect of PD, we performed the GO function and KEGG pathway by using the R language and Bioconductor software package. In the GO enrichment analysis results, the top 10 elements were picked from biological process (BP), cellular component (CC), and molecular function (MF) for visual analysis (Figure 6). In the BP items, the anticolon cancer effect of PD is mainly concentrated in response to drug, response to oxidative stress, reactive oxygen species, metallic process, response to molecular of bacterial origin, response to reactive oxygen species, response to metal ion, response to lipopolysaccharide, response to oxygen levels, response to steroid hormone, and cellular response to chemical stress. The MF project mainly includes nuclear receptor activity, DNA-binding transcription factor binding, G protein-coupled amine receptor activity, ligand activated, steroid hormone receptor activity, ubiquitin-like protein ligase binding, RNA polymerase II-specific DNA-binding transcription factor binding, catecholamine binding, transcription factor activity, drug binding, and ubiquitin–protein ligase binding. A variety of biological processes also show that PD could be utilized to treat colon cancer and other illnesses.

**FIGURE 7:**
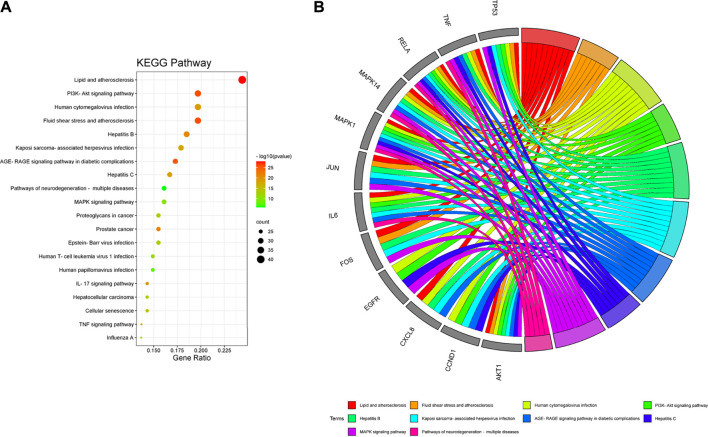
Kyoto Encyclopedia of Genes and Genomes (KEGG) pathway enrichment analysis of PD in the treatment of colon cancer. **(A)** bubble diagram of KEGG pathway enrichment. **(B)** GO chord diagram of the top 10 pathways in PD anticolon cancer.

**FIGURE 8:**
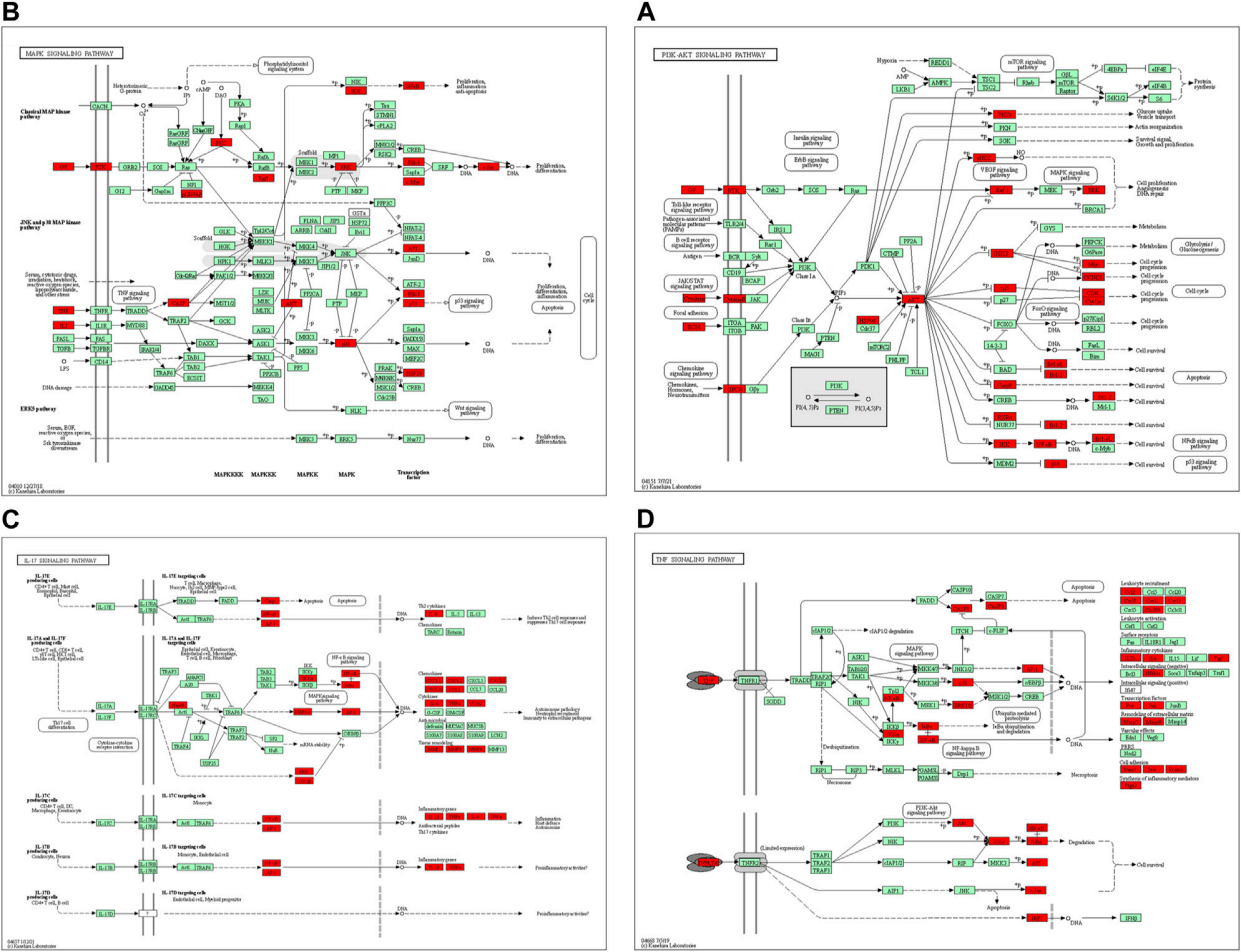
The primary pathways were colored using the KEGG mapper. Red nodes represent the targets regulated by PD in colon cancer. **(A)** PI3K-Akt signaling pathway. **(B)** MAPK signaling pathway. **(C)** IL-17 signaling pathway. **(D)** TNF signaling pathway.

In the KEGG pathway analysis (Figure 7), the size of the node indicates the number of targets converged to the pathway, and the greater the node, the greater the quantity of targets enriched. The color of the node ranges from green to red indicates the *p*-value from large to small, so the larger the red node indicates the higher the significance of the signaling pathway and the more important the role, which mainly includes lipid and atherosclerosis, PI3K-Akt signaling pathway, fluid shear stress and atherosclerosis, AGE-RAGE signaling pathway in diabetic complications, hepatitis B, cellular senescence, MAPK signaling pathway, pathways of neurodegeneration-multiple diseases, human cytomegalovirus infection, Epstein–Barr virus infection, proteoglycans in cancer, human T-cell leukemia virus 1 infection, human papillomavirus infection, prostate cancer, IL-17 signaling pathway, hepatocellular carcinoma, TNF signaling pathway, influenza A, Kaposi sarcoma-associated herpesvirus infection, and hepatitis C. We found that many enrichment pathways are associated with additional pathological effects, possibly caused by homologous molecular targets in various diseases. Among them, the PI3K-Akt signaling pathway, MAPK signaling pathway, IL-17 signaling pathway, TNF signaling pathway, cellular senescence, and proteoglycans in cancer were most closely associated with the mechanism of PD anticolon cancer (Figure 8).

### 3.5 Molecular docking findings

**FIGURE 9:**
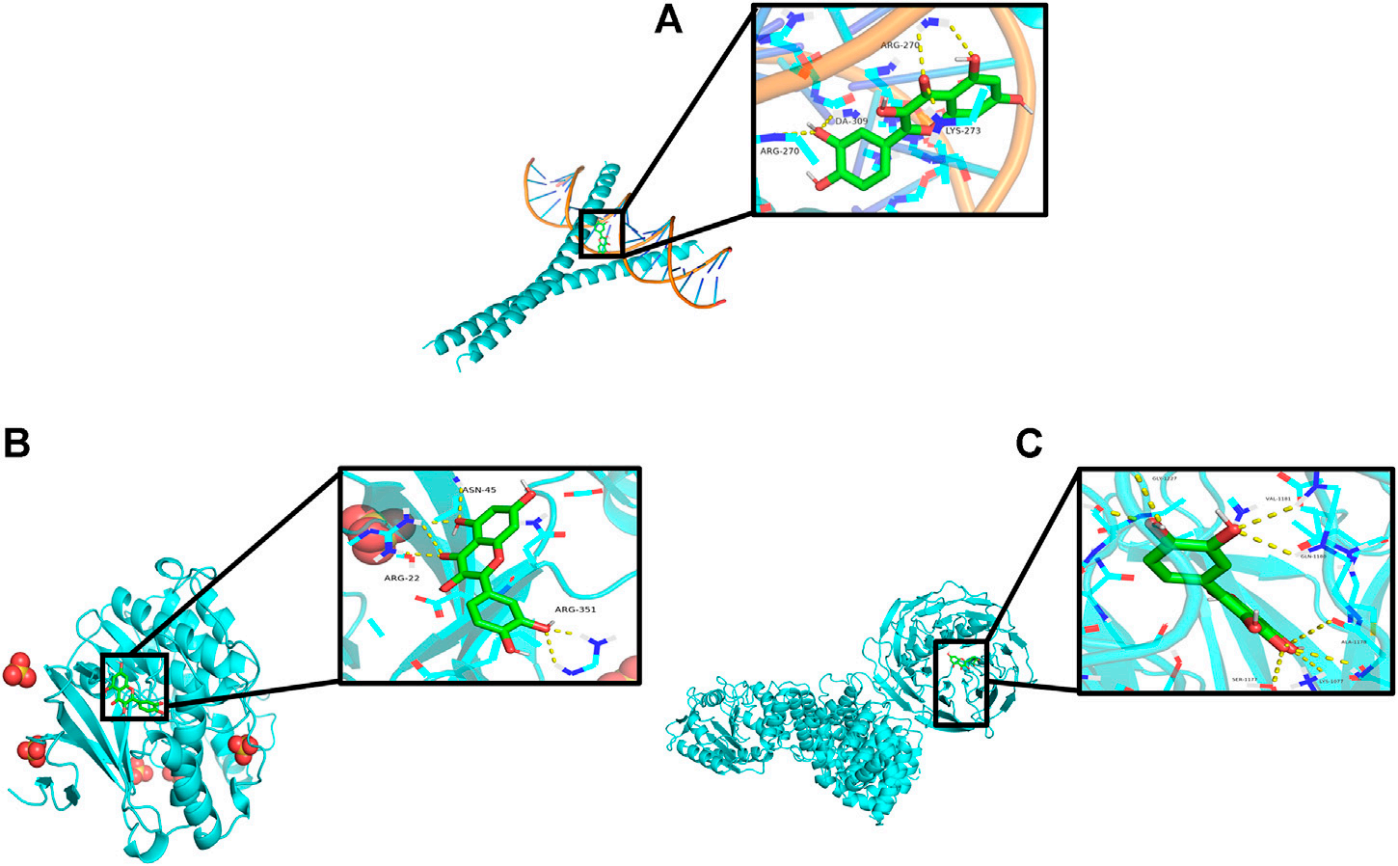
Schematic diagram of quercetin docking with core targets. **(A)** JUN docking with quercetin. **(B)** MAPK1 docking with quercetin. **(C)** AKT1 docking with quercetin.

The “Components–Targets–Pathway” network diagram revealed that the quercetin node is the main active component of PD. Topological analysis shows that MAPK1, JUN, and AKT1 were the main targets. Therefore, the molecular docking of MAPK1, JUN, and AKT1 with quercetin was carried out by using Auto Dock Vina software. The results are shown in Figures 9A–C. An index of ≤ −5.0 kcal/mol (Xiang et al., 2022), which represents two molecules with a standard binding capacity, is used. It can be found that quercetin interacts with MAPK1 through three amino acid residues, namely, ASN-45, ARG-22, and ARG-351, with a hydrogen bonding energy of −5.0 kcal/mol; with JUN through three amino acid residues, namely, ARG-270, DA-309, and LYS-273, with a hydrogen bonding energy of −8.9 kcal/mol; and with AKT1 through six amino acid residues, namely, GLY-1227, VAL-1181, GLN-1180, ALA-1178, SER-1177, and LYS-1077, with a hydrogen bonding energy of −9.0 kcal/mol.

## 4 Discussion

Colon cancer is a common malignant neoplastic disease that often occurs at the intersection between the sigmoid colon and colon. Its pathogenesis is complex. Smoking, alcohol consumption, red meat consumption, and family history of colon cancer are all considered to be the risk factors for this cancer (Young et al., 2010; Chan et al., 2011; Fedirko et al., 2011). With the gradual increase in morbidity rate in recent years and the high tumor recurrence rate in some patients after surgery, the health and quality of life of patients have been seriously and adversely affected (Mejri et al., 2017; Ramphal et al., 2018; Gupta et al., 2019). Issues like side effects and tolerance to anticolon cancer medications have led researchers to reconsider new alternatives to treat colon cancer. Research shows that TCM has unique advantages in cancer treatment, such as high efficiency and fewer side effects (Liu et al., 2020). Because of the complexity of TCM components, many TCM components have been proven to have certain curative effects in the laboratory (Qi et al., 2015). Individual TCM component has been shown to be effective in the treatment of tumor diseases, but the results of studies on TCM compound component are relatively few. However, the TCM treatment of diseases mostly appears in the form of a whole, so the research of TCM compounds for the treatment of illnesses needs to be further confirmed (Ling et al., 2014; Liu et al., 2019; Wang J. et al., 2020).

With the advancement of modern science and technology, medical research is becoming more and more important with the help of computer technology and other research tools. As new research technology means integrating system biology, bioinformatics, and network science, network pharmacology can analyze the molecular association between drugs and therapeutic objects and reveal the systemic pharmacological mechanism of drugs from the overall perspective of systemic level and biological network. Its characteristics of multichannel and multitarget are very suitable for research with this kind of complex component of TCM (Societies, 2021). Therefore, this study systematically revealed the mechanism of PD in the treatment of colon cancer.

According to the “Components–Targets–Pathway” network, quercetin, isorhamnetin, and beta-sitosterol may be the most important potential components of PD anticolon cancer. It has been reported that quercetin can bind to kinases such as PI3K-Akt/PKB, SEK1, and MAPK to induce tumor cell cycle arrest and inhibit growth and metastasis, antioxidant replication, and angiogenesis by participating in the induction and expression of phosphorylation of key intracellular targets (Darband et al., 2018). Cosan et al. (2011) demonstrated the mechanism of quercetin anticolon cancer by targeting telomerase activity and inhibiting Akt phosphorylation to induce cell senescence. Li C et al. pointed out that isorhamnetin inhibited the proliferation of SW480, HCT116, and HT-29cells by inhibiting the PI3K-Akt-MTOR signaling pathway; reduced the phosphorylation level of Akt, phosph-p70s6 kinase, and phosph-4e-bp1 (T37/46) proteins; and enhanced the expression of Cyclin B1 protein, thereby inhibiting the progression of colon cancer cells (Li et al., 2014). Suho S et al. verified the antioxidant effect of isorhamnetin, which proved its antimetastasis of colon cancer cells by inhibiting ROS-mediated HIF1α accumulation (Seo et al., 2016). Beta-sitosterol can reduce β-catenin and proliferating cell nuclear antigen with proliferative activity in colon cancer cells, to regulate the apoptosis and multiplication in tumor cells (Bin Sayeed and Ameen, 2015). Furthermore, beta-sitosterol can regulate the p53/NF-κB/BCRP signal axis to adjust the response of colorectal cancer to chemotherapy (Wang Z. et al., 2020). In addition, there are some biological processes of other active components of PD anticolon cancer, which need to be further explored.

The PPI network analysis of the duplicate targets of PD and colon cancer revealed that the core targets were MAPK1, JUN, AKT1, TP53, TNF, RELA, MAPK14, CXCL8, ESR1, and FOS. There are abundant interactions with other targets, indicating a higher degree of hub, to complete specific molecular functions. It was demonstrated that MARK1 and MAPK14 can affect the multiplication and apoptosis in cancer cells (Fang and Richardson, 2005; Madkour et al., 2021). Yong Y et al. significantly inhibited the invasion and metastasis in colon tumor cells by interfering with the expression of MAPK1 (Yang et al., 2018). Felipe PM et al. discovered that MAPK14 expression was higher in colorectal tumors (Wang D. et al., 2022) and that tumor cell progression and apoptosis could be induced by inhibiting MAPK14 expression in colon cancer samples (Chiacchiera and Simone, 2008). Ryan W S et al. verified that JUN could bind to and participate in the KRAS-mediated transcriptional activation of the USP28 promoter, followed by KRAS inducing transcriptional silencing of the repressor gene TSG to affect colon cancer cells (Serra et al., 2014). Overexpression of Akt can be detected in the early stages of colon cancer (Roy et al., 2002). Diminished Akt signaling, inhibition of phosphorylation processes, and downregulation of expression are implicated in the differentiation, apoptosis, and metastasis of colon cancer cells (Agarwal et al., 2013; Minnee and Faller, 2021). Colorectal carcinogenesis is closely associated with TP53 gene mutations, which can be a trigger for cancer, and its high expression in colorectal cancer is often indicative of poor survival (Li et al., 2018; Li L. et al., 2020a). TNF can cause apoptosis and is also an inflammatory cytokine (Dash et al., 2021). Inflammation contributes to tumor development, and overproduction of the pro-inflammatory factor TNF can both induce tumor cell progression (Ma et al., 2017; Cruceriu et al., 2020). Chen T et al. detected that by preventing REAL deacetylation, RELA acetyl-dependent expression was promoted, which in turn affected the migration and invasion of colon tumor cells (Chen et al., 2017). The expression of TNF-β and TNF-α and their receptors can induce the activation of NF-κB and NF-κB-related genes, thus regulating the survival, metastasis, etc. of colon tumors (Buhrmann et al., 2019).

The results of GO enrichment analysis showed that the anticolon cancer effect of PD may be related to a variety of biological processes, mainly including response to molecule of bacterial origin, response to oxidative stress, response to reactive oxygen species, response to lipopolysaccharide, cellular response to chemical stress, G protein-coupled amine receptor activity, membrane raft, drug binding, steroid hormone receptor activity, ubiquitin-like protein ligase binding. Moreover, the results reflect the involvement of multiple changes in cell proliferation, apoptosis, energy metabolism, signal transduction, and immune regulation in the process of PD against colon cancer.

The results of KEGG enrichment analysis indicated that numerous different illnesses were also enriched besides colorectal cancer, probably because of the existence of the same molecular targets in different disease pathologies processes. We selected signaling pathways closely connected with colorectal cancer for the analysis. We found that “PI3K-Akt signaling pathway,” “MAPK signaling pathway,” “Proteoglycans in cancer,” “IL-17 signaling pathway,” “Cellular senescence,” and “TNF signaling pathway” may be the potentially critical mechanism of PD anticolon cancer. The PI3K-Akt signaling pathway is an important signal transduction pathway for colon tumors. PI3K activation produces phosphatidylinositol triphosphate, which then leads to the activation of Akt through a series of signaling cascades. Akt activation can inhibit or activate a series of downstream substrates through phosphorylation, further regulating cell differentiation, proliferation, migration, and apoptosis (Danielsen et al., 2015). Activation of PI3K/Akt signal significantly promoted β-catenin nuclear translocation, cell proliferation, and apoptosis in colorectal cancer epithelial cells (Bishnupuri et al., 2019). Huang et al. (2020) found that different concentrations of quercetin could downregulate the protein levels of PI3K, Akt, p-Akt, and BCL2 and upregulate the protein level of Bax, to inhibit the proliferation and induce apoptosis of HCT116 colon cancer cells and HT29 colon cancer cells through PI3K-Akt signaling pathway. Further studies showed that quercetin inhibited the activity of Akt by blocking the phosphorylation level of Akt, thus regulating colon cancer through the PI3K-Akt signaling pathway (Refolo et al., 2015; Yang et al., 2016; Raja et al., 2017). Alterations in the MAPK signaling pathway are strongly associated with the progression of colon cancer cells (Burotto et al., 2014). Xu C et al. demonstrated that altering the expression of ERK1/2 and Akt genes downstream of the MAPK signaling pathway regulates DNA synthesis, proliferation, and apoptosis in colon cancer cells (Xu C. et al., 2022). Ma W et al. downregulated the phosphorylated expression of MEK1/2, ERK1/2, and RAF1 in the MAPK signaling pathway as a way to regulate the malignant proliferating phenotype of colon tumors (Ma et al., 2021). Wang Z et al. used Baicalin to upregulate the decidual protein induced by progesterone to activate the Ras and Raf signals to induce senescence in human colon cancer cells, (Wang et al., 2018b). It was found that quercetin could upregulate JNK, c-Jun, p-P38, and P53 and downregulate p-ERK in the MAPK signaling pathway, to promote apoptosis of colon cancer Caco-2, DLD-1, and HCT-15 and cause SW620 cell cycle arrest (Dihal et al., 2008; Kim et al., 2013; Refolo et al., 2015; Kee et al., 2016; Zhang et al., 2017). IL-17 signaling pathway and TNF signaling pathway may be mainly involved in inflammation and immune regulation. Dong P et al. inhibited the proliferation of colon cancer cells and reduced inflammatory cytokines by inhibiting the expression of IL17f mRNA in the IL-17 signaling pathway (Pan et al., 2022). Xun W et al. pointed out that the IL-17 signal pathway and TNF signal pathway regulate PD-L1 expression in colorectal cancer HCT116 cells, and the expression of PD-L1 is closely related to colon cancer treatment (Wang et al., 2017; Yaghoubi et al., 2019). Proteoglycans are complex molecule existing in the extracellular matrix and cell membrane, which plays an important role in cell growth, metastasis, and invasion. Yip g et al. found that the imbalance of the expression of biosynthesis and degradation enzymes of heparan sulfate proteoglycan can affect various stages of tumorigenesis (Yip et al., 2006). Zang YQ et al. promoted cancer progression by targeting the miR200 family with lumican to promote epithelial cell transformation into mesenchymal cells. Therefore, the regulation of proteoglycan expression is an important approach to treating colorectal cancer (Zang et al., 2021). Cellular senescence is a state of growth arrest, and induction of cellular senescence can inhibit the development of cancer (Sun et al., 2007). Hou Z et al. downregulated the expression of p21 through the interaction between Tribbles homolog 2 and AP4 to inhibit cellular senescence and thus regulate colon tumor cell progression (Hou et al., 2018). With the above results, it was considered that PD against colon cancer achieves its effects through various pathways such as tumor metabolic pathway, apoptotic pathway, and inflammatory pathway. In addition, many other signaling pathways have been shown, and their mechanisms of action need to be further explored.

## 5 Conclusion

Our study utilized network pharmacology theory and relevant software to systematically elaborate the potential mechanism of PD anticolon cancer. A variety of components of PD could exert an effective role in the treatment of colon cancer through multiple pathways and multiple targets. The accuracy of the outcomes was further verified using subsequent molecular docking. The results showed that PD could regulate the proliferation, metastasis, differentiation, senescence, apoptosis, and other biological processes of tumor cells by regulating the expression of related genes in colon cancer cells, reflecting the anticolon cancer effect of PD. However, the research results still need to be further corroborated using animal experiments and other relevant experiments to ensure the reliability of the study results. In summary, our research provides a new basis for further exploration and subsequent experimental verification of PD in the treatment of colon cancer.

## Data Availability

The original contributions presented in the study are included in the article/Supplementary Material; further inquiries can be directed to the corresponding authors.
